# Compressive Behavior of Aluminum Microfibers Reinforced Semi-Rigid Polyurethane Foams

**DOI:** 10.3390/polym10121298

**Published:** 2018-11-23

**Authors:** Emanoil Linul, Cristina Vălean, Petrică-Andrei Linul

**Affiliations:** 1 Department of Mechanics and Strength of Materials, Politehnica University of Timisoara, 1 Mihai Viteazu Avenue, 300222 Timisoara, Romania; cristina_valean@yahoo.com; 2 Faculty of Industrial Chemistry and Environmental Engineering, Politehnica University of Timisoara, 6 Vasile Parvan Avenue, 300223 Timisoara, Romania; linulpetrica@yahoo.com; 3 National Institute of Research for Electrochemistry and Condensed Matter, Aurel Paunescu Podeanu Street 144, 300569 Timisoara, Romania

**Keywords:** semi-rigid polyurethane foams, aluminum microfibers, quasi-static compression tests, mechanical properties, energy absorption capability

## Abstract

Unreinforced and reinforced semi-rigid polyurethane (PU) foams were prepared and their compressive behavior was investigated. Aluminum microfibers (AMs) were added to the formulations to investigate their effect on mechanical properties and crush performances of closed-cell semi-rigid PU foams. Physical and mechanical properties of foams, including foam density, quasi-elastic gradient, compressive strength, densification strain, and energy absorption capability, were determined. The quasi-static compression tests were carried out at room temperature on cubic samples with a loading speed of 10 mm/min. Experimental results showed that the elastic properties and compressive strengths of reinforced semi-rigid PU foams were increased by addition of AMs into the foams. This increase in properties (61.81%-compressive strength and 71.29%-energy absorption) was obtained by adding up to 1.5% (of the foam liquid mass) aluminum microfibers. Above this upper limit of 1.5% AMs (e.g., 2% AMs), the compressive behavior changes and the energy absorption increases only by 12.68%; while the strength properties decreases by about 14.58% compared to unreinforced semi-rigid PU foam. The energy absorption performances of AMs reinforced semi-rigid PU foams were also found to be dependent on the percentage of microfiber in the same manner as the elastic and strength properties.

## 1. Introduction

Porous materials (PMs), such as polymeric [1,2,3], metallic [4,5,6], and ceramic [7,8] foams, have been widely spread in recent years to a variety of engineering applications due to their exceptional mechanical, physical, thermal, and acoustic properties. The main properties of the foam materials (FMs) have a direct connection with the size (cell-wall thickness and cell length), shape (from regular to the most irregular shapes), and topology (connections between cells) of the cells that constitute the PMs. Regardless of the matrix constituent (polymeric, metallic, or ceramic material), cellular materials (CMs) are ideal energy absorbers. This feature of the FMs is highlighted by the appearance of a large flat/hardening plateau region (up to 70% strain) at almost constant stress [9,10,11].

Polymeric foams (PFs) are a promising category of CMs because they can be obtained at a relatively low cost compared to the other kind of FMs. The PFs show many engineering applications depending on their physical properties. Because of their very low thermal conductivity, one of the main uses of PFs is like a thermal insulator for modern buildings, refrigerated trucks/railway cars, ships designed to carry liquid natural gas, pipes, etc. [12,13]. Contrary to fully dense solid materials [14,15], the PFs are non-corrosive in a damp salt-water environment, so they are widely used in marine applications (rafts and floatation devices) [16,17,18]. In addition, open-cell FMs are used as filters at many different levels, as water-repellent membranes that allow air to permeate whatever is underneath the membrane, or even as a hydrophobic barrier in some high-quality sporting and leisurewear [16,17,19]. PFs, especially polyurethane (PU) foams, are used in the sport, automotive, and medical industries to absorb energy, and to reduce sound/noise and vibrations [20,21].

In recent years, different techniques have been developed for manufacturing flexible [22,23] and rigid [24,25,26] PMs with closed, open, or mixed (partly open and partly closed) cells. Also, the effect of different reinforcements (particles, fibers, etc.) on the mechanical and physical properties of PFs was studied in previous works. Soto and co-workers [27] presented a route for the production of more environmentally friendly filled flexible PU foams through the replacement of part of the synthetic polyol by biobased ones, and by the addition of waste tire particles. Good acoustic absorption properties were found by the authors in a wide range of frequencies. Short glass-fibers, glass micro-spheres, and chopped glass-fiber strands were used by Khanna and Gopalan [16] to reinforce polyurethane flexible foam. The authors observed that short glass fibers are more effective in improving the tensile and flexural deformation response of the foam compared to other reinforcing fillers. All types of the reinforced foams show degradation in compressive strength compared to the unfilled polyurethane foams. Gama and co-workers [28] evaluated the sound absorption properties of rigid polyurethane foams produced from crude glycerol (CG) and/or liquefied coffee grounds derived polyol (POL). The POL derived foam has slightly higher sound absorption coefficient values at lower frequencies, while the CG foam has higher sound absorption coefficient values at higher frequencies. The influence of potato protein (PP) on the rigid polyurethane foams’ morphology and on physical and mechanical properties were explored by Członka and co-workers [29]. The authors show that an addition of 0.1 wt % PP improves the compressive behavior, while the addition of PP over a certain optimal level has a negative effect on the physico-mechanical properties. Rigid polyurethane foams reinforced with buffing dust (BD) were characterized by Członka and co-workers [30] by means of mechanical and thermal methods. Depending on the amount of BD in polymer mixture, resulting composites exhibit improvement or deterioration of abovementioned properties. Patricio and co-workers [31] studied the effect of poly lactic acid (PLA) addition into poly (e-caprolactone) (PCL) matrices on the morphological, thermal, chemical, mechanical and biological performance of the 3D constructs produced with a novel biomanufacturing device. Their results show that the addition of PLA to PCL scaffolds strongly improves the biomechanical performance of the constructs, compared to blends prepared by melt blending.

Flexible and rigid polyurethane foams have found limited applications in the transport industry for design of vehicle lightweight composite structures in terms of increased crash energy resistance [32]. On the one hand, flexible PU foams are used on a large scale for cushioning and vibration damping, but they are worse in terms of impact energy absorption performances [33]. On the other hand, rigid PU foams shows good energy absorption capabilities, but they are too rigid and present plastic collapse from a much earlier stage of deformation [34]. The most useful foam would be one that presents a combination of the best properties of the two mentioned PU foams. Therefore, this paper proposes a methodology for obtaining reinforced semi-rigid polyurethane foams using aluminum microfibers in a polymeric matrix. The effect of aluminum microfibers on the main mechanical properties and energy absorption capability is investigated. The obtained semi-rigid PU foams highlight a higher load bearing capacity with appropriate energy absorption performances, elastic properties, and compression strength. 

## 2. Materials and Methods

### 2.1. Materials

**Figure 1:**
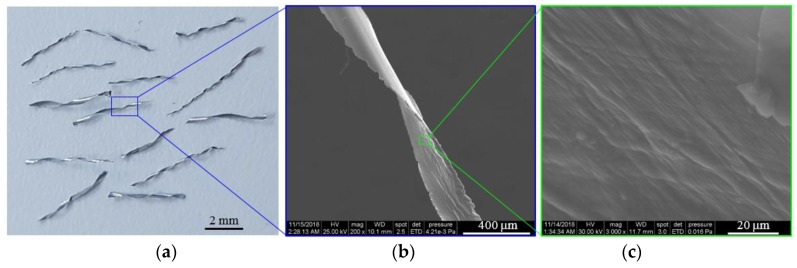
Optical (**a**) and SEM images (**b**, **c**) of AMs.

Unreinforced and reinforced closed-cell semi-rigid polyurethane (PU) foams with a density of 0.15 g/cm^3^ were prepared in the laboratories of the National Institute of Research for Electrochemistry and Condensed Matter (Timisoara, Romania). The polymer matrix was made up of polyol (200 mL) and isocyanate (180 mL), while aluminum solid wastes (referred to in the paper as aluminum microfibers) were reinforcements. The aluminum microfibers (AMs) shows a repetitive geometric shape and the foam manufacturing acting as the recycling process. Figure 1 presents the optical and SEM images of used AMs.

The used AMs had a length of 4–6 mm and a cross section of 270 ± 40 μm (width) × 37 ± 3 μm (thickness). The chemical composition of the commercially available AMs is shown in Table 1.

**Figure 2:**
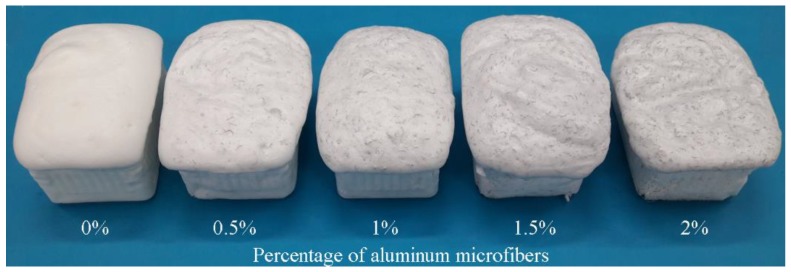
Semi-rigid PU foam blocks obtained depending on the percentage of AMs.

The collection and insertion of the AMs into the foam matrix material was done following a well-established procedure. Semi-rigid PU foams with different contents of the aluminum microfibers (0, 0.5, 1, 1.5, and 2% AMs of the foam liquid mass) were prepared using a two-step method. Firstly, the AM were added half to the isocyanate solution and half to the polyol solution, followed by an individual mechanically stirred process for 3 min to ensure their complete homogenization. Before being added to the individual components, the AMs were dried at 80 °C for about 60 min. Secondly, after the individual stirring process, the two components (isocyanate and polyol together with the corresponding percentage of reinforcements) were mixed and mechanically stirred together for 30 s. The obtained reinforced PU foam was dried in a controlled environment, at room temperature (25 °C), for 24 h [35]. After the drying and hardening process, large semi-rigid PU foam blocks were obtained (see Figure 2). The same procedure (less reinforcement) was followed to obtain unreinforced PU foams. The foam density was measured using both mass and sample dimensions. The average resulting foam density was 0.15 g/cm^3^ and the samples with a density above or below the 10% range were excluded [36].

The reaction parameters (percentage of foam components, time, temperature, etc.) were optimized in order to produce the most economical and functional reinforced semi-rigid PU foam [37]. The resulting PU foams were marked as U-PU foam (unreinforced semi-rigid PU foam) and R-PU foam (reinforced semi-rigid PU foam).

### 2.2. Methods

**Figure 3:**
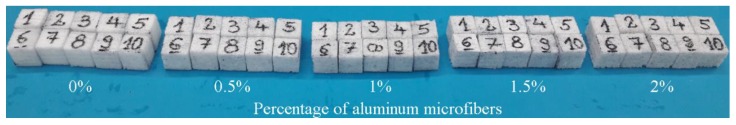
Semi-rigid PU foam blocks obtained depending on the percentage of AMs.

Uniaxial quasi-static compression tests were carried out on a 5 kN Zwick Roell 005 testing machine (ZwickRoell LP, Kennesaw, GA, USA). The experimental tests were performed on cubic samples (22.5 mm × 22.5 mm × 22.5 mm); using a constant crosshead speed of 10 mm/min. Ten samples were provided for each test condition and the properties of the semi-rigid PU foams were determined according to ASTM D1621-16 standard [38] (see Figure 3). 

The compressive properties were investigated in a direction parallel to the free direction of the foam rise at a maximum load of about 300 N. The material properties were assessed in the controlled room temperature and humidity conditions on samples taken from the center of the foam blocks.

## 3. Results and Discussions

**Figure 4:**
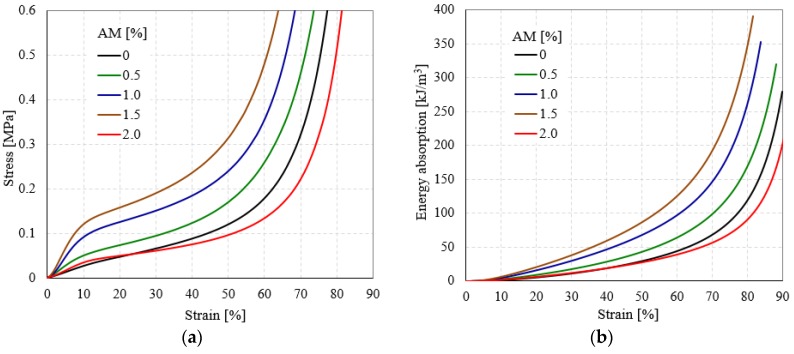
Compressive engineering stress-engineering strain (**a**) and energy absorption-engineering strain (**b**) curves of semi-rigid PU foams.

Quasi-static compressive tests were carried out to investigate the main mechanical properties of the semi-rigid PU foams, since they also play an important role in the energy absorption performances and can be of high interest for possible applications in automotive, sport, and building construction industries [39,40,41]. Figure 4 presents the compressive engineering stress (σ)–engineering strain (ε) and energy absorption (W)-strain (ε) curves for unreinforced and AMs reinforced semi-rigid PU foams.

Regardless of semi-rigid PU foam type (unreinforced or reinforced), each foam sample is characterized by similar quasi-static compression behavior, exhibiting three different regions: A narrow linear-elastic region (< 5% strain), followed by a stress-plateau region (around 10–40%), and ending with a densification region (over 40% strain) [42,43,44]. 

As is well known, the limited slope of the linear elastic area from the stress-strain curves is directly related to the foam compression modulus [45,46,47]. The σ-ε curve of unreinforced and reinforced semi-rigid PU foams exhibit a smooth transition from the linear to the plateau region. In this case, there is no well-defined yield point corresponding to the compressive yield strength because there is no drop stress [48,49,50]. This behavior is typical of semi-rigid and flexible PU foams, which differ significantly from that of rigid foams [51,52]. After the elastic-plateau transition area, the σ-ε curves exhibit an extended strain hardening plateau region outstanding in the field of energy absorption. In this region, the main foam collapse mechanisms occur [53,54]. With an increasing content of AMs of the foam liquid mass, the investigated foams exhibit a shorter range of elongation (measured up to a predetermined stress) because of the gradual loss of PU matrix flexibility. In terms of the densification strain, R-PU foams show lower values than unreinforced ones, indicating that the samples can sustain slightly lower deformation without collapsing. 

The main quasi-static compressive mechanical properties (quasi-elastic gradient, 0.2% offset yield stress, 1% offset yield stress, plateau stress, densification strain, and energy absorption at densification strain) of the unreinforced and reinforced semi-rigid PU foams modified with aluminum microfibers are reported in Table 2. The investigation of elastic properties was based only on compression loading tests, while unloading tests were not considered [55]. Densification strain is defined as the strain at which the slope of the curve in a plot of energy efficiency versus strain is zero. The densification strain of cellular materials represents the start of the cell-wall interactions, which enhance the compressive resistance of a cellular solid [56].

The volumetric energy absorption capacity, *W*, of investigated PU foams is defined by Equation (1), and by using variable integration limits, it can be interpreted as the area under the engineering stress-engineering strain curves [57,58].

(1) $$\;W=\int_{0}^{\varepsilon }\sigma d\varepsilon \;$$

The energy absorption values at different strains (10, 20, 30, 40, 50, 60, 70, and 80% engineering strain) of investigated foams are presented in Table 3.

**Figure 5:**
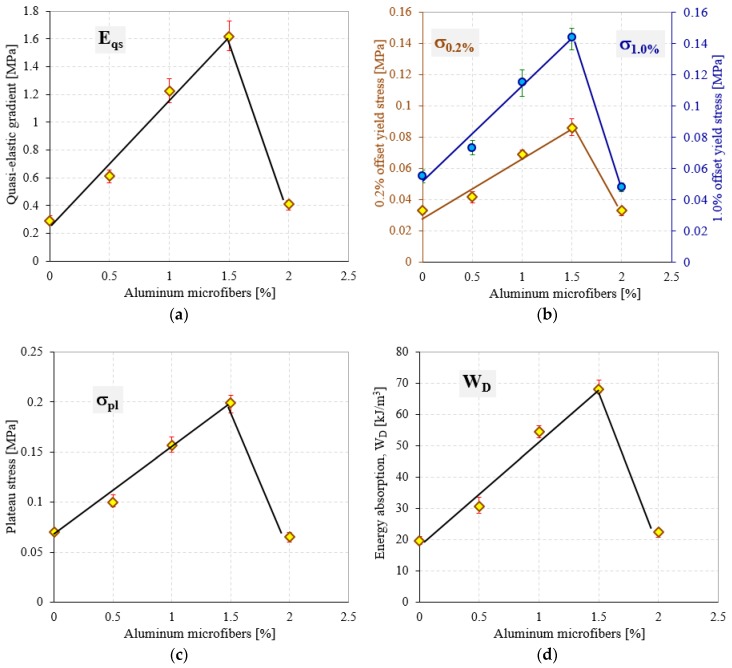
Mechanical properties of investigated semi-rigid PU foams: (**a**) Quasi-elastic gradient; (**b**) 0.2% and 1.0% offset yield stress; (**c**) plateau stress; (**d**) energy absorption at densification strain.

Comparing the data from Table 2 and the variation of properties shown in Figure 5, it can be denoted that the investigated mechanical properties of the modified semi-rigid PU foams increase as aluminum microfibers content increases. This behavior is attributed to the rigidity of the aluminum microfibers’ structure, which introduced more cross-links in the PU foam network. Notice should be made that this increase in mechanical performances was obtained by adding up to a certain limit of AMs. Above this upper limit, the quasi-static compressive behavior changes and the mechanical properties decrease significantly, exhibiting values almost equal to U-PU foam. Furthermore, the W capabilities of R-PU foams were also found to be dependent on the percentage of AMs in the same manner as the elastic and strength mechanical properties (see Figure 4b and Table 3).

**Figure 6:**
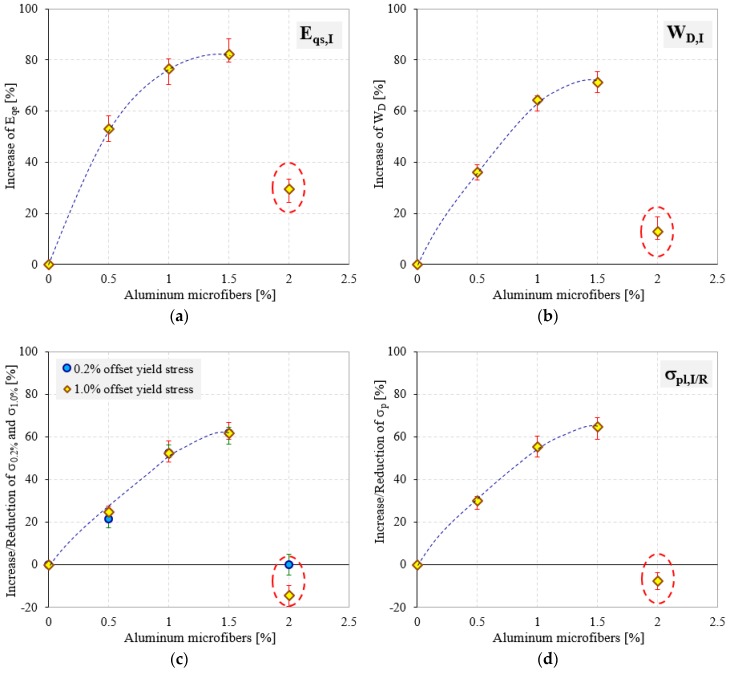
Percentage increase/decrease of the quasi-elastic gradient (**a**); energy absorption (**b**); 0.2 and 1% offset yield stresses (**c**) and plateau stress (**d**) for modified semi-rigid PU foam samples normalized by reference PU foam (0% AMs).

An analysis of the elastic results presented in Figure 5a indicates that the quasi-elastic gradient (*E*_qe_) of semi-rigid modified PU foams significantly increases with the increase in the percentage of AMs in their cellular structure. Therefore, considering the normalized data, the presence of aluminum microfibers results in an increase in foam stiffness up to about six times relative to U-PU foam. In addition, considerable increases in the case of strength properties (0.2 and 1% offset yield stresses and plateau stress) have also been observed. These increases in properties were obtained by adding in the liquid mass of the PU foam up to a maximum of 1.5% aluminum microfibers. In contrast, the addition of 2% AMs to the foam liquid mass leads to a decrease in mechanical properties up to 75% compared to 1.5% AMs reinforced foams, as shown in Table 2 and Figure 6. It seems that the effect of the AMs in the foams modified with 2 wt % is less significant, probably due to the kinetic reactions occurring between the liquid reactive mixture (isocyanate and polyol) and aluminum microfibers. This effect leads to a decrease of the growth rate of the foam formation (increased viscosity) and a less homogeneous foam structure, and at the same time, to more unstable failure mechanisms.

Figure 6 shows the percentage increase/decrease values of mechanical properties of the modified semi-rigid PU foams (0.5, 1.0, 1.5, 2% AMs) normalized by U-PU foam (0% AMs). 

From Figure 6, it is obvious that the reinforcements have a significant and useful effect for increasing the mechanical properties of the semi-rigid PU foams. It has been found that reinforcing the foams with maximum 1.5% AMs leads to an increase in elastic properties of up to 82.20%. In addition, the increase percentage of energy absorption and strength properties is about 71.29% (for W) and 61.81% (for 1% offset yield stress). Compared with R-PU foams up to 1.5% AMs, the PU foams reinforced with 2% AMs do not show significant increases of their quasi-static compressive properties with respect to the U-PU foam. Quasi-elastic gradient shows a percentage increase of up to 29.41%, while energy absorption capacity increases by only 12.68% (Figure 6a,b). Furthermore, a negative effect on the strength properties (0.2 and 1% offset yield stresses and plateau stress) of 2% AMs reinforced foams was observed. In this case, the 1% offset yield stress showed a reduction percentage of 14.58% and a plateau stress of 7.69%.

## 4. Conclusions

In the present investigation, aluminum microfibers (AMs) and a polymer matrix (made up of polyol and isocyanate) were used to produce reinforced semi-rigid polyurethane (PU) foams. Therefore, a low-density closed-cell semi-rigid PU foam with a density of 0.15 g/cm^3^ was obtained. Quasi-static compressive tests were performed on cubic samples to investigate the mechanical properties of the produced foams. It has been observed that the presence of AMs has a direct effect on the main properties of the reinforced foams. The experimental results indicate that with increasing AMs content into the foam matrix, the PU foams are characterized by higher compressive strength (about 61.81%) and energy absorption performances (about 71.29%). However, larger AMs filler contents (2 wt %) do not lead to further changes in energy absorption capability (around 12.68%), highlighting even a negative effect on the strength properties (about 14.58%).

## Figures and Tables

**Table 1:** Chemical composition of AMs.

Element	Al	Si	Fe	Cu	Mn	Mg	Cr	Zn	Ti	Other
wt.%	Balance	0.7–1.3	0.50	0.10	0.4–1.0	0.6–1.2	0.25	0.20	0.10	0.15

**Table 2:** The main compressive mechanical properties of investigated semi-rigid PU foams.

AMs (%)	Quasi-Elastic Gradient (MPa)	0.2% Offset Yield Stress (MPa)	1% Offset Yield Stress (MPa)	Plateau Stress (MPa)	Densification Strain (%)	Energy Absorption ^1^ (kJ/m^3^)
0	0.288 ± 0.03	0.033 ± 0.001	0.055 ± 0.004	0.070 ± 0.003	43.901 ± 0.86	19.53 ± 1.05
0.5	0.614 ± 0.04	0.042 ± 0.003	0.073 ± 0.004	0.100 ± 0.006	43.382 ± 0.52	30.54 ± 1.59
1.0	1.222 ± 0.09	0.069 ± 0.002	0.115 ± 0.009	0.157 ± 0.008	41.381 ± 0.71	54.47 ± 1.29
1.5	1.618 ± 0.11	0.086 ± 0.005	0.144 ± 0.007	0.199 ± 0.008	40.510 ± 0.93	68.02 ± 1.37
2.0	0.408 ± 0.03	0.033 ± 0.002	0.048 ± 0.003	0.065 ± 0.005	44.140 ± 0.64	22.36 ± 1.04

^1^ Energy absorption values at densification strain.

**Table 3:** The mean energy absorption values of investigated PUF foams at different strains (kJ/m^3^).

AMs (%)	10%	20%	30%	40%	50%	60%	70%	80%
0	1.51 ± 0.18	5.48 ± 0.39	11.30 ± 0.44	19.53 ± 0.88	29.59 ± 0.75	44.36 ± 1.26	68.40 ± 1.21	119.16 ± 2.11
0.5	2.85 ± 0.41	9.32 ± 0.55	17.86 ± 0.47	28.84 ± 0.76	43.49 ± 0.84	64.70 ± 1.26	99.53 ± 1.63	170.89 ± 2.35
1.0	4.83 ± 0.50	16.13 ± 0.48	30.10 ± 0.92	46.96 ± 0.98	68.13 ± 1.13	97.36 ± 1.34	146.70 ± 1.92	261.46 ± 3.19
1.5	6.54 ± 0.45	20.92 ± 0.67	38.43 ± 0.78	59.75 ± 0.83	87.04 ± 1.05	125.79 ± 1.66	192.84 ± 2.72	347.67 ± 4.64
2.0	1.89 ± 0.40	6.41 ± 0.46	12.11 ± 0.61	19.07 ± 0.63	27.76 ± 0.99	39.27 ± 1.15	56.64 ± 1.27	90.72 ± 1.79
